# Agnosia for bird calls

**DOI:** 10.1016/j.neuropsychologia.2018.03.024

**Published:** 2018-05

**Authors:** Louwai Muhammed, Chris J.D. Hardy, Lucy L. Russell, Charles R. Marshall, Camilla N. Clark, Rebecca L. Bond, Elizabeth K. Warrington, Jason D. Warren

**Affiliations:** Dementia Research Centre, UCL Institute of Neurology, University College London, London, United Kingdom

**Keywords:** Bird, Auditory agnosia, Semantic dementia, Semantic category

## Abstract

The cognitive organisation of nonverbal auditory knowledge remains poorly defined. Deficits of environmental sound as well as word and visual object knowledge are well-recognised in semantic dementia. However, it is unclear how auditory cognition breaks down in this disorder and how this relates to deficits in other knowledge modalities. We had the opportunity to study a patient with a typical syndrome of semantic dementia who had extensive premorbid knowledge of birds, allowing us to assess the impact of the disease on the processing of auditory in relation to visual and verbal attributes of this specific knowledge category. We designed a novel neuropsychological test to probe knowledge of particular avian characteristics (size, behaviour [migratory or nonmigratory], habitat [whether or not primarily water-dwelling]) in the nonverbal auditory, visual and verbal modalities, based on a uniform two-alternative-forced-choice procedure. The patient's performance was compared to healthy older individuals of similar birding experience. We further compared his performance on this test of bird knowledge with his knowledge of familiar human voices and faces. Relative to healthy birder controls, the patient showed marked deficits of bird call and bird name knowledge but relatively preserved knowledge of avian visual attributes and retained knowledge of human voices and faces. In both the auditory and visual modalities, his knowledge of the avian characteristics of size and behaviour was intact whereas his knowledge of the associated characteristic of habitat was deficient. This case provides further evidence that nonverbal auditory knowledge has a fractionated organisation that can be differentially targeted in semantic dementia.

## Highlights

• The cognitive organisation of auditory semantics is poorly understood.
• We assessed multimodal avian knowledge in a birder with semantic dementia.
• The patient had auditory (but not visual) agnosia for birds versus healthy birders.
• Auditory knowledge of avian attributes and human voices were differentially affected.
• This case illuminates the fractionated organisation of nonverbal auditory knowledge.

## 1 Introduction

The cognitive organisation of knowledge about nonverbal sounds remains poorly understood. This is attributable in part to a lack of detailed neuropsychological models of nonverbal auditory semantics and also the comparative rarity of reports of selective auditory agnosia, which might reveal the critical underlying cognitive architecture (Engelien et al., 1995, Clarke et al., 2000, Hattiangadi et al., 2005, Saygin et al., 2010, Slevc and Shell, 2015). It has been proposed that the processing of sounds as ‘auditory objects’ may be organised analogously to visual object processing, with corresponding neural mechanisms in auditory cortex and its connections in the temporal, parietal and frontal lobes (Goll et al., 2010a, Goll et al., 2010b, Brefczynski-Lewis and Lewis, 2017). However, opportunities to resolve key issues in auditory cognition – based on the study of patients with relevant deficits – remain limited.

One such important issue concerns the extent to which nonverbal auditory knowledge is differentiated. In the visual modality, it is relatively well established that object recognition is hierarchical (encompassing different levels of knowledge, ranging from general and superordinate to more specific and fine-grained) and categorical (knowledge about different kinds of objects having cognitive and neural substrates that are at least partly separable) (Warrington, 1975, Jefferies and Lambon Ralph, 2006, Lambon Ralph et al., 2010, McCarthy and Warrington, 2016). The extent of differentiation within the visual semantic system is modulated by experience, as illustrated by the effects of brain damage in individuals possessing specific expertise with certain object categories (such as plants or cars: Jefferies et al., 2011). It is not clear whether similar cognitive organisational principles apply to auditory objects. In the case of one specialised semantic domain – knowledge about familiar people – impaired auditory recognition (phonagnosia) selective within the auditory modality and between modalities has been documented, to set alongside the better known visual equivalent of prosopagnosia (Hailstone et al., 2010, Hailstone et al., 2011, Luzzi et al., 2017). Specific agnosias have also been described for the equally specialised auditory domain of music (Ayotte et al., 2000, Clark et al., 2015). Studies of patients with auditory agnosia following focal brain damage have suggested that recognition of environmental sounds may dissociate from other kinds of auditory information processing (Engelien et al., 1995, Clarke et al., 2000, Hattiangadi et al., 2005, Saygin et al., 2010). However, a more fine-grained analysis of environmental sound recognition has remained largely elusive, in part because knowledge about sounds is generally not graded comparably with voices or melodies and often lacks a precise equivalent in other sensory modalities. Further key issues in semantic cognition concern the extent to which different input modalities contribute to multimodal or amodal conceptual representations and the representation of unique entities (Warrington, 1975, McCarthy and Warrington, 1988; Lambon Ralph et al., 2010; Wong and Gallate, 2012; Lambon Ralph, 2013; Gainotti, 2017). Here again, nonverbal sound is potentially an important test case, since most data have been gathered for the verbal and visual routes to semantic knowledge.

We recently had the opportunity to address these issues concerning the cognitive organisation of nonverbal sound knowledge in a patient, BA, with semantic dementia (SD) who possessed unusual premorbid expertise in the domain of bird knowledge. In addition to its resonance in clinical neurology as a leading focal brain degeneration of younger life (Hodges and Patterson, 2007, Warren et al., 2013), SD has played a pivotal role in the development of our current understanding of the human semantic system (Warrington, 1975, Lambon Ralph et al., 2010, Lambon Ralph, 2013, McCarthy and Warrington, 1988, McCarthy and Warrington, 2016). Semantic dementia is a highly coherent clinic-anatomical syndrome; it typically presents as a fluent progressive aphasia led by loss of vocabulary but typically evolves to multimodal impairment of conceptual and object knowledge (Hodges and Patterson, 2007, Lambon Ralph, 2013), underpinned by selective disintegration of the neural networks that mediate semantic processing. These networks are centred on the anterior temporal lobes and semantic dementia is characteristically associated with anterior temporal lobe atrophy, usually more marked in the left cerebral hemisphere (Hodges and Patterson, 2007, Fletcher and Warren, 2011). Impaired recognition of sounds is well attested in SD (Bozeat et al., 2000, Goll et al., 2010a, Goll et al., 2010b, Hsieh et al., 2011, Hailstone et al., 2011, Golden et al., 2015). Indeed, SD constitutes a unique crucible for exploring the cognitive organisation of auditory and other nonverbal knowledge systems.

While the processing of voices and melodies in SD has been analysed at some length (Omar et al., 2010, Hailstone et al., 2011, Hsieh et al., 2011, Golden et al., 2015), the organisation of environmental sound processing in this disorder has not been delineated in like detail. BA's special knowledge of birds is therefore particularly apposite: birds constitute a semantic domain that is finely graded (comprising a multiplicity of species with defining features superimposed on basic superordinate avian characteristics) and comparably accessible via the auditory and visual sensory modalities. The impact of semantic dementia on bird recognition should therefore expose the cognitive organisation of a highly differentiated category of environmental sound knowledge, and allow direct comparisons between knowledge modalities and with other specialised knowledge domains (such as human voices). Moreover, experienced bird enthusiasts (‘birders’) generally acquire knowledge of bird appearance and bird calls in tandem; it should therefore be possible to disambiguate the effects of acquired expertise from intrinsic modality-specific contributions to this knowledge category. In studying BA, we designed a neuropsychological experiment to probe different dimensions of avian knowledge relating to avian physical features and associated ethological characteristics, in parallel via auditory (bird call), visual (bird appearance) and verbal (bird name) input modalities. We created novel stimuli to assess knowledge of the same bird attributes via each of these input channels independently: conventional tests of semantic processing often rely on cross-modal matching tasks that involve two or more processing channels (e.g., names and pictures), however such tests confound interpretation of modality-specific effects. BA's performance on our novel semantic test was referenced to a control group of healthy older individuals with similar birding experience. In addition, we sought to compare BA's knowledge of birds with his knowledge of familiar people via their voices and faces, in order to assess the specificity of any avian semantic deficit.

## 2 Methods

### 2.1 Clinical details of patient BA

BA is a right-handed male former credit union executive aged 65 who presented with a six year history of progressive loss of vocabulary. He had particular difficulty finding the names of people and objects and would ask the meaning of words such as ‘spatial’. His conversation had become increasingly imprecise but remained fluent, with no history of speech sound errors or dysarthria. He was less inclined to read newspapers and magazines due to difficulty understanding their text. His general intellect was well preserved, including memory for recent autobiographical events and facility with financial transactions and household gadgets. His family considered that he had become a little more emotionally labile but there had been no significant behavioural or personality change and in particular, no instances of social disinhibition or faux pas. There was no past medical history of note nor any relevant family history. He had undergone pure tone audiometry on account of his impaired speech comprehension; this revealed only mild age-related hearing loss.

BA is a dedicated amateur birder with some 30 years’ experience, including around 10 weeks each spring spent in birdwatching expeditions and over the years had also regularly attended courses in bird call recognition, visual identification and bird behaviour. He had extensive exposure to a range of bird species representing all major regions and habitats of the British Isles. He had noted waning of his ability to name birds or identify them from their calls over a similar timeframe to his evolving difficulty with general vocabulary. At the time of assessment, he was also becoming less competent at identifying birds visually but he continued to enjoy recognising and feeding the birds that visited his garden. There had been no suggestion of any difficulty recognising familiar faces or household items nor any difficulty recognising the voices of telephone callers or everyday noises. There had been no evident change in BA's appreciation of music.

On examination his speech was circumlocutory with impoverished content but grammatically correct and normally articulated. He exhibited impaired picture naming and single word comprehension; repetition of polysyllabic words and phrases, arithmetic, praxis and visuoperceptual functions were intact. General neuropsychological assessment supported the bedside impression of severe anomia underpinned by a selective, primary semantic memory deficit, with associated deficits of episodic verbal and face memory but intact speech production, sentence processing, executive skills and auditory and visual perceptual functions in the context of very superior performance IQ (Table 1). The general neurological examination was normal.

**Table 1:** General neuropsychological findings in patient BA.

**Cognitive domain**	**Score**	**Control reference**
General intellect		
MMSE (/30)	29	N/A
WASI Verbal IQ	**87**	N/A
WASI Performance IQ	131	N/A
Executive skills		
WMS-R Digit Span Reverse (max)	7	> 95th %ile
Stroop D-KEFS color naming (s)	20	> 50th %ile
Stroop D-KEFS word naming (s)	15	> 50th %ile
Stroop D-KEFS ink color naming (s)	41	> 50th %ile
Trails Part A (s)	23	> 50th %ile
Trails Part B (s)	55	> 50th %ile
Episodic memory		
RMT Faces (/50)	**28**	< 5th %ile
RMT Words (/50)	**35**	5th %ile
Short-term memory		
WMS-R Digit Span Forward (max)	8	> 95th %ile
Parietal skills		
GDA Total (/24)	22	> 50th %ile
VOSP Object Decision (/20)	17	25–50th %ile
BST (/30)	23	70th %ile
Auditory perceptual processing		
PALPA-3 (/36)	36	35.5 (32–36)
Confrontation naming		
GNT (/30)	**0**	< 1st %ile
BNT (/30	**2**	29.2 (28–30)
Comprehension		
BPVS (/150)	**111**	148 (145–150)
Concrete synonyms (/25)	**20**	10th %ile
Abstract synonyms (/25)	**15**	2nd−5th %ile
PALPA-55 (/24)	24	23.8 (22–24)
Repetition		
Words (/45)	45	44.6 (43–45)
Nonwords (/20)	20	18.1 (12–20)
Sentences (/10)	10	9.7 (8–10)
Agrammatism		
Spoken sentence construction (/25)	25	25.0 (25–25)
Written sentence construction (/25)	25	24.9 (24–25)

The raw scores obtained by BA on each test are shown with maximum scores in parentheses for each neuropsychological test unless otherwise indicated. Percentile (%ile) equivalents for raw scores are indicated where published norms are available; scores at or below the 10th percentile on standardized tests are indicated in **bold**. Scores on tests for which published norms are not available are referenced to a local cohort of 20 healthy age-matched control subjects in the format mean (range). Note that we used a reduced 30-item version of the BNT. Key: BNT, Boston Naming Test; BPVS, British Picture Vocabulary Scale; BST, Baxter Spelling Test; D-KEFS, Delis-Kaplan Executive Function System; GDA, Graded Difficulty Arithmetic Test; GNT, Graded Naming Test; MMSE, Mini-Mental State Examination; N/A, not applicable; PALPA, Psycholinguistic Assessments of Language Processing in Aphasia (this subtest assesses phoneme discrimination on spoken syllable pairs); RMT, Recognition Memory Test; Warrington Synonyms Tests; Trails A/B; VOSP, Visual Object and Space Perception; WASI, Wechsler Abbreviated Scale of Intelligence; WMS-R, Wechsler Memory Scale-Revised.

**Fig. 1:**
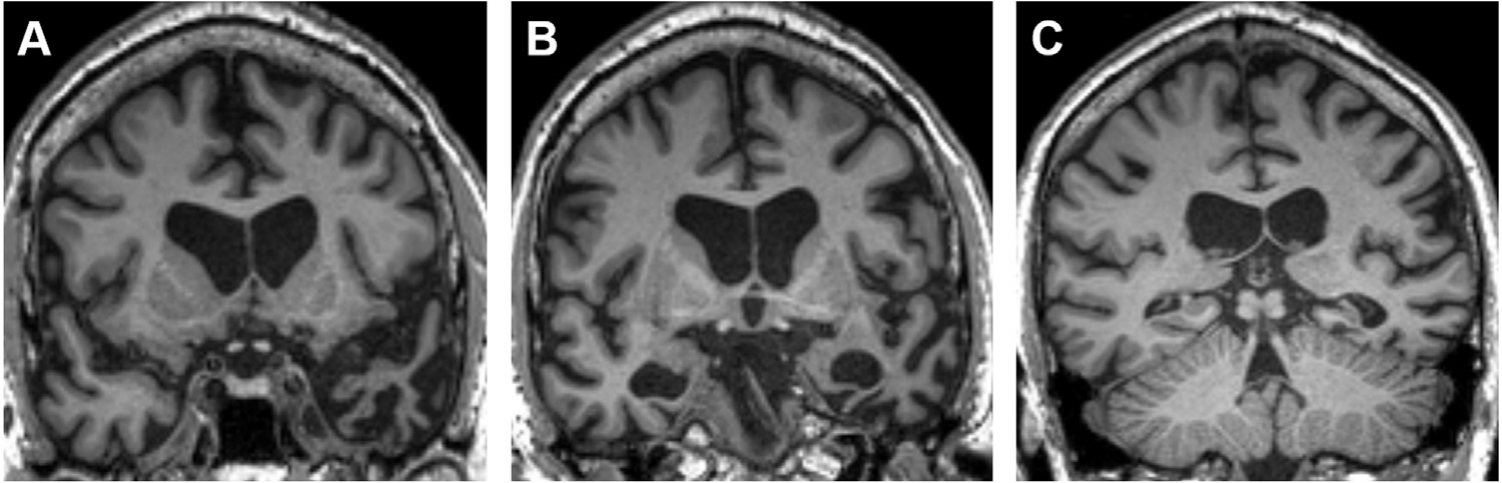
Brain MRI findings in patient BA. Coronal sections of BA's T1-weighted volumetric brain MRI through the temporal poles (A), mid-anterior temporal lobes (B) and temporo-parietal junctional zones (C) show asymmetric, focal temporal lobe atrophy typical of semantic dementia. There is more severe involvement of the left temporal lobe (projected here on the right) and within each temporal lobe, marked atrophy of the pole, inferior temporal cortex and mesial temporal structures, with relative sparing of superior temporal gyrus and more posterior cortices.

Brain MRI (Fig. 1) showed asymmetric atrophy predominantly affecting the anterior, mesial and inferior temporal lobes, more marked on the left. Together with the clinical presentation and neuropsychological findings, the MRI appearances were typical of SD, as defined in current consensus diagnostic criteria (Gorno Tempini et al., 2011).

### 2.2 Healthy control participants

Three older amateur birders with no history of neurological or otological disease also participated in the experimental study. These healthy control participants were members of BA's birding group and had similar birding experience, such as attending the same fieldtrips and educational activities; their details are summarised in Table 2.

**Table 2:** Summary of experimental findings in BA and healthy birder controls.

**Characteristic**	**BA**	**Control 1**	**Control 2**	**Control 3**	**Control group** mean (range)
Age	64	57	56	70	61
Gender	M	M	F	F	–
Handedness	R	R	R	R	–
Birding experience (yrs)	30	40	9	11	20
BIRD KNOWLEDGE:					
Auditory (calls) (/96)^a^	**69**	88	82	82	84 (82 – 88)
*Size? (/32)*^*b*^	*29*	*30*	*28*	*31*	*29.7 (28 – 31)*
*Migratory? (/32)*^*b*^	*22*	*29*	*26*	22*	*25.7 (22 – 29)*
*Water-dwelling? (/32)*^*b*^	***18***	*29*	*28*	*29*	*28.7 (28 – 29)*
Visual (pictures) (/96)^a^	87	91	85	91	89 (85 – 91)
*Size? (/32)*^*b*^	*31*	*31*	*29*	*29*	*29.7 (29 – 31)*
*Migratory? (/32)*^*b*^	*27*	*28*	*25*	*30*	*27.7 (25 – 30)*
*Water-dwelling? (/32)*^*b*^	***29***	*32*	*31*	*32*	*31.7 (31 – 32)*
Verbal (names) (/96)^a^	**70**	93	94	93	93.3 (93 – 94)
*Size? (/32)*^*b*^	***24***	*32*	*32*	*31*	*31.7 (31 – 32)*
*Migratory? (/32)*^*b*^	***19***	*29*	*30*	*30*	*29.7 (29 – 30)*
*Water-dwelling? (/32)*^*b*^	***27***	*32*	*32*	*32*	*32*
PERSON KNOWLEDGE:					
Auditory (voices) (/48)^c^	37	N/A	N/A	N/A	41.5 (35 – 46)**
Visual (faces) (/48)^c^	41	N/A	N/A	N/A	46.6 (41 – 48)**

Maximum scores on experimental tests are indicated in parentheses; scores obtained by BA falling outside the healthy control range for that test are indicated in bold. a, chance score = 48; b, chance score = 16; c, chance score = 24; F, female; M, male; N/A, not available; R, right-handed; *this control participant's score on this subtest is attributable to a low score on one block of 9 trials – excluding this block in all participants yields the following scores: BA 68% (unchanged), Control 1 95% (improved), Control 2 91% (improved), Control 3 77% (improved; i.e., BA performs inferiorly to all controls on the reanalysed subtest but the overall profile of his results on the auditory experiment is unchanged); **data from historical group (Hailstone et al., 2011) of 35 healthy controls (mean age 63.9 (5.7) years, 13 male).

The study was approved by the local institutional ethics committee and all participants gave informed consent in accordance with the guidelines of the Declaration of Helsinki.

### 2.3 Experimental test design and stimuli

#### 2.3.1 Assessment of bird knowledge

**Fig. 2:**
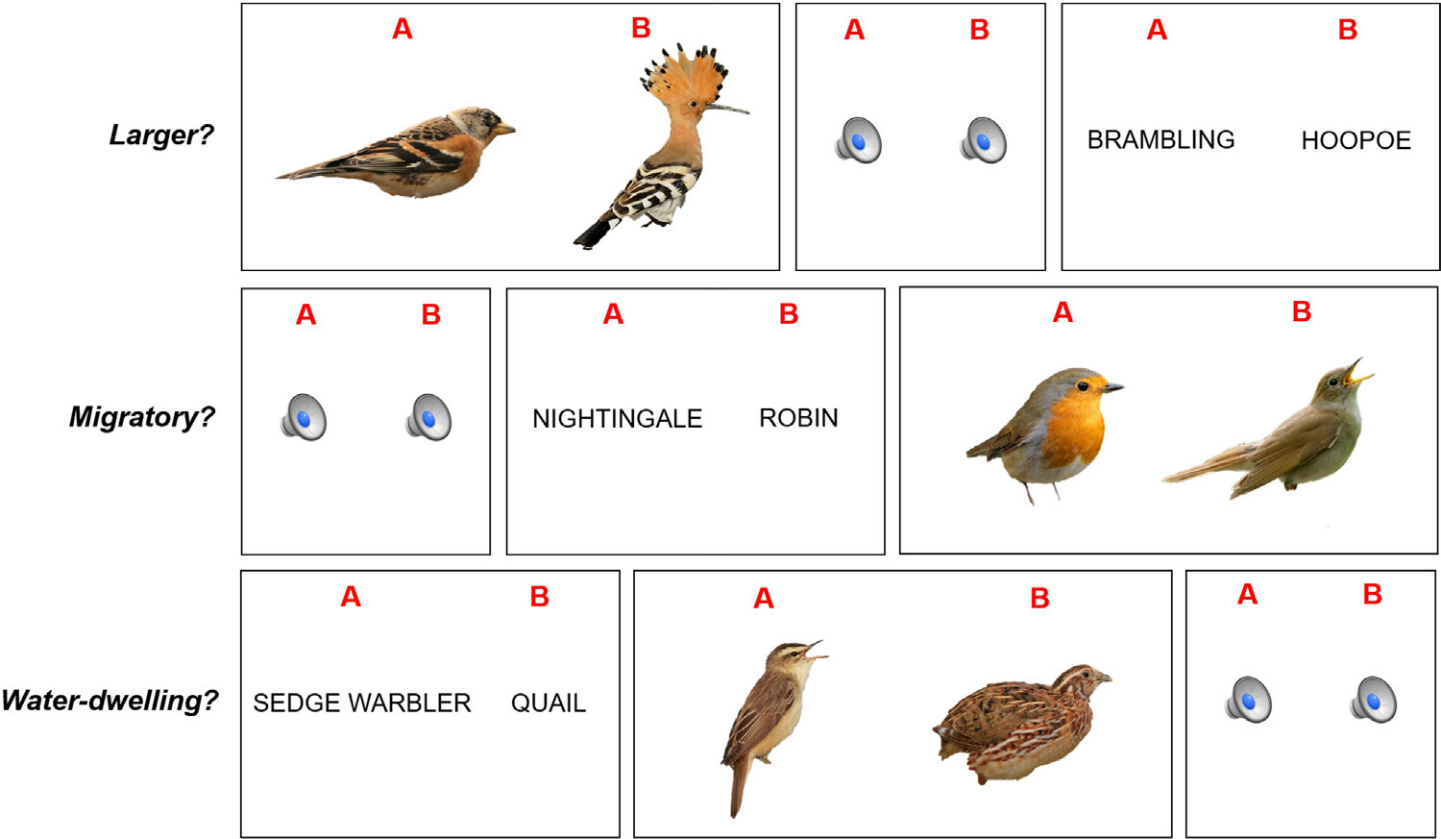
Examples of stimulus trials used in the experimental test of bird knowledge. The test was designed to probe different modalities (auditory, visual, verbal) of bird knowledge and different avian semantic characteristics (size, behaviour, habitat) via each modality. On each trial, the task was a single forced choice decision on a pair of stimuli (bird pictures, written names or sequentially presented call sounds), differentiated according to the nominated characteristic; the participant was required to indicate which of the two birds represented (A or B) was larger (size decision; here, the target is ‘Hoopoe’), which was a migrant (behaviour decision; here, the target is ‘Nightingale’) or which would generally be found near water (habitat decision; here, the target is ‘Sedge warbler’). On each trial, target and foil bird species were matched for the irrelevant characteristics (e.g., for the size decision here, both the hoopoe and brambling are non-water-dwelling migrants). Modalities and characteristics were presented in interleaved blocks of trials such that there was no net preferential ordering and the relative positions of targets and foils were fully randomised between trials.

In order to assemble a suitable set of bird stimuli, we first identified 64 British bird species likely to be familiar to an experienced amateur birder in the UK, based on the 2015 Royal Society for the Protection of Birds national survey and consultation with the London Bird Club. This list of birds was chosen to vary three key avian characteristics, representing different dimensions of bird knowledge: physical (size), behaviour (whether or not migratory) and habitat (whether or not primarily dwelling near water). As we intended to probe these dimensions via the auditory modality, we further chose bird species that had identifiable calls as well as visual attributes. All 64 birds in the list were classified according into one of eight categories (e.g., *larger – migratory – water-dwelling*…), balancing for all three nominated semantic characteristics across the stimulus set; the complete categorised list of stimulus species is presented in Table S1 in Supplementary Material on-line. We then obtained pictures and sound recordings representing the visual appearance and the call of each bird species. Pictures were derived by searching Google Image for high-resolution photographs of each bird (typically shown standing in profile); using Powerpoint^®^, the images were edited to uniform dimensions and to remove backgrounds and the birds’ feet (since webbed feet would present a common, basic visual cue to a watery habitat; see examples in Fig. 2). Bird call recordings were derived as MP3 files from on-line databases (http://www.xeno-canto.org; http://www.british-birdsongs.uk) using GarageBand^®^, recordings were edited to fix mean intensity and duration (a five second section of each recording was selected for each bird, including at least one complete, typical phrase in species with more extended calls and free of intrusive extraneous bird calls, water sounds or other background noises; examples are in Supplementary Material on-line). Although it was not assessed explicitly as an experimental variable, the emotional value of the bird call stimuli was also indexed using pleasantness ratings from four older healthy individuals without birding experience who did not participate in the main experiment (details in Table S1).

To create a multimodal test of bird knowledge, we arranged these bird stimuli into three subtests probing knowledge derived respectively within the auditory, visual and verbal (written common name) modalities. Bird calls, bird pictures and bird names were paired to form individual trials in the corresponding auditory, visual and verbal subtests. On each trial, one characteristic (size, behaviour or habitat) was varied between the paired stimuli while other characteristics were fixed. Examples of trials for each subtest are presented in Fig. 2. Each subtest comprised 96 trials in toto (three sets of 32 trials, each varying one of the three characteristics). To minimise any confounding effect of stimulus ordering, blocks of 10 or 11 trials representing each modality (auditory, visual or verbal) and avian characteristic (size, behaviour or habitat) were delivered interleaved in a double Latin square design and the order of trials was randomised within each block. The task on each trial was to decide which of the two bird call, picture or name stimuli had the nominated characteristic (larger, migratory or water-dwelling). These avian characteristics are not absolute: the key design principle of the test (and the basis for the semantic decision on each trial) lay in the comparison of the bird species comprising each stimulus pair (i.e., we defined species attributes relative to one another; for example, on trials requiring a size decision, the members of each stimulus pair were chosen such that one bird species was unambiguously larger than the other).

#### 2.3.2 Assessment of person knowledge

In order to assess BA's performance in another highly differentiated semantic domain (person knowledge), we used the test of voice and face familiarity previously described by Hailstone et al. (2011). For each modality, 48 trials (24 famous people, 24 unfamiliar people) were presented in randomised order. For this test, famous voices and faces were selected based on a pilot survey of healthy older British people; familiar and unfamiliar trials were balanced for age and gender and voice stimuli were free of verbal identifiers. The task on each trial was to decide whether the voice or face was familiar or unfamiliar. Voices were presented first (in order to minimise priming effects in the vocal modality), followed by faces. BA's performance on this test was referenced to an historical cohort of 35 healthy older controls (Hailstone et al., 2011).

### 2.4 General experimental test procedure

Stimuli were presented via a notebook computer in a quiet room. Before the experiment, practice trials (using stimuli not presented during the test proper) were administered to ensure that BA and the healthy control participants understood the task instructions. During the test, no feedback was given about performance and no time limits were imposed. The test was administered in two divided sessions with an intervening rest period and additional short rest periods were offered between blocks of trials. Participant responses were recorded for offline analysis.

## 3 Results

The performance profiles of BA and healthy controls on the experimental tests of bird and person knowledge are summarised in Table 2.

### 3.1 Bird knowledge

The healthy controls collectively achieved high scores on all three (auditory, visual and verbal) modalities of the bird knowledge test. Control performance was near-ceiling and most consistent for bird knowledge derived from names, relatively weaker and more variable for knowledge derived from appearance and weakest for knowledge derived from calls. Across modalities, controls showed excellent knowledge of a physical avian characteristic (size) and knowledge of habitat (whether or not water-dwelling) superior to knowledge of behaviour (whether or not migratory).

In terms of his overall performance by modality, BA performed below the control range for bird knowledge derived from calls and names but within the control range for knowledge derived from appearance. Examining his performance for particular semantic characteristics, via the verbal modality BA's knowledge of bird size, behaviour and habitat were all impaired relative to controls, though knowledge of behaviour was relatively more impaired. Via the auditory and visual modalities, BA's knowledge of bird size and behaviour were within the control range while knowledge of bird habitat was impaired; referenced to the healthy control profile, BA's deficit of bird habitat knowledge was clearly more severe via the auditory than the visual modality, and comparably severe via the auditory and verbal modalities.

### 3.2 Person knowledge

BA performed within the healthy control range for the tests of famous voice and face knowledge. In line with the healthy control profile, he performed somewhat better when assessing the familiarity of faces than voices.

## 4 Discussion

We have shown that a highly differentiated domain of auditory knowledge – bird calls – can be degraded in SD, despite relative preservation of visual knowledge within this domain (bird appearance) and relative preservation of knowledge of familiar human voices and faces. BA performed well above chance in all modalities and (across modalities) for all avian semantic characteristics (size, behaviour and habitat), and he achieved a very creditable score in the visual modality, arguing that he understood the task (which was the same across modalities and characteristics). It is unlikely that his retained performance in the visual modality can be ascribed simply to special ornithological expertise, given that an experienced birder acquires similar facility in recognising bird names and call sounds and these other modalities were clearly impaired in BA. Nor can BA's agnosia for bird calls be ascribed to a more general impairment in processing semantic exemplars, given his performance on the tests assessing person knowledge was within the range of healthy older controls. BA's performance profile further argues against implicit verbal recoding of the nonverbal stimuli presented here: BA's verbal semantic capacity was inferior both to his visual semantic capacity and to his nonverbal auditory semantic capacity for the avian attributes of size and migratory behaviour. It is unlikely that BA had any significant deficit of auditory perceptual processing: he achieved normal performance both on a standard test of phoneme discrimination (see Table 1) and for one nonverbal auditory semantic subtest (assessing the characteristic of avian size, Table 2). We interpret the profile of BA's results, taken together, as indicative of a relatively selective, associative nonverbal auditory agnosia.

These findings have implications for the organisation of the auditory semantic system. BA's clearly superior knowledge of birds via the visual modality compared with the auditory modality implies that these two modalities constitute separable routes to avian conceptual knowledge. Such a separation is consistent with functional neuroimaging evidence in the healthy brain concerning other domains of nonverbal knowledge (Martin, 2007, Gainotti, 2017). A general issue for inferences of this kind arises from the difficulty of balancing processing demands between modalities: many sensory objects are much more readily apprehended by sight than by sound (or the converse), and the relative familiarity of particular objects across modalities is often a challenging factor to control. We argue that avian knowledge constitutes a privileged domain in this respect, since many birds have salient calls as well as visual features and experience in these two modalities tends to be acquired together. Disproportionate degradation of one channel in SD (as here) can then be parsimoniously ascribed to an underlying modularity of the underlying cognitive or neural mechanisms. The findings in BA do not strongly support the dissociation of verbal and nonverbal auditory processing, since (as anticipated) his recognition of bird names was also substantially impaired relative to the healthy control birders. However, verbal and nonverbal mechanisms are likely to be separable (McCarthy and Warrington, 1988, Engelien et al., 1995, Saygin et al., 2010, Gainotti, 2017) and the profile of BA's performance for particular avian semantic characteristics tentatively supports this: inspecting Table 2, BA showed deficits across all semantic characteristics via the verbal modality but a more discrete pattern via the nonverbal auditory modality.

Given that (referenced to healthy controls) BA did not exhibit a deficit of familiar voice recognition, his agnosia for bird calls further implies that the brain mechanisms that underpin these categories of auditory knowledge are separable. Human voices and bird calls share certain cognitive similarities: both represent classes of auditory objects with multiple conjoined features and recognition of such objects depends on distinguishing their integrated featural representations from many other similar objects (Goll et al., 2010a, Goll et al., 2010b, Brefczynski-Lewis and Lewis, 2017). This is most strikingly illustrated by human voices, which constitute exemplars unique to particular individuals. BA's superior performance for recognition of human over avian voices is therefore unlikely to reflect the relative extent of object differentiation within these semantic domains: rather, this dissociation implies dedicated processing modules for different categories of nonverbal auditory knowledge, in line with current fractionated neuropsychological models of human voice and environmental sound processing (Goll et al., 2010a, Goll et al., 2010b, Slevc and Shell, 2015).

It is noteworthy that knowledge of an avian physical characteristic (size) was uniformly well preserved via both the auditory and visual modalities in BA and in the healthy control birders, whereas knowledge of other avian characteristics was more variable in the healthy controls and dissociated in BA (see Table 2). We propose that knowledge of avian size rests on certain fundamental ‘templates’ (for example, more elongated bodies, relatively longer legs and longer vocal tracts in larger birds) that could be used to achieve superordinate recognition of this physical attribute in both the auditory and visual modalities. On the other hand, knowledge of avian behaviour and habitat rely on more arbitrary associations that are less closely tied to physical features intrinsic to the species; the webbed foot, a relatively reliable feature of water birds, was removed here while there are few, if any, reliable physical predictors of migratory behaviour in British birds. It is tempting, however, to speculate that BA's superior knowledge of avian behaviour versus habitat may be attributable at least in part to cueing from episodic memory: observation of migratory (unlike water) birds is necessarily seasonal. Within the verbal modality, both BA and the healthy controls evidently performed particularly well when attributing habitat to names – this is likely, at least in part, to reflect the embedding of broadly ‘aquatic’ cues into the common names of water birds (‘reed bunting’, ‘oystercatcher’, ‘sandpiper’, etc - though note also ‘woodcock’ versus ‘turtle dove’; see Table S1).

We argue that the findings in BA's case are compatible with emerging formulations of the cognitive pathophysiology of SD. Progressive erosion of the hierarchical and multidimensional systems that assign meaning to sensory objects is a fundamental consequence of SD and leads typically to initial loss of more fine-grained and arbitrary associations, followed by knowledge of superordinate and generic features. The profile of deficits shown by BA in both the auditory and visual modalities conforms to a sequence of this kind. While the panmodal nature of the defining semantic lesion in SD has been emphasised, it has also been recognised that semantic deficits in SD may be relatively modality-selective (Warrington, 1975, McCarthy and Warrington, 1988, Gainotti, 2017). Our findings here do not speak directly to the issue of semantic integration of sensory object concepts or the existence of a multimodal or amodal ‘hub’ (Lambon Ralph, 2013): rather, the findings suggest that the semantic input channels to such higher order integrative mechanisms via particular sensory modalities are differentially vulnerable in SD. From a clinical perspective, this work suggests that it may be time to incorporate tests of auditory recognition into the assessment of patients with SD, if indeed sounds are a more sensitive index of nonverbal semantic integrity than the picture stimuli that are more conventionally employed.

This study has several limitations. To determine the true selectivity of BA's agnosia within the auditory modality would require a more detailed assessment of auditory apperceptive processing and semantic processing of other categories of sounds. In particular, it would be of interest to assess his ability to recognise familiar melodies, particularly given the interesting formal and cognitive similarities of birdsong to human music (and indeed, speech: Shannon, 2016). This parallel analysis could potentially extend to judgements on emotional content, as bird calls (like melodies, but unusually for a specific class of environmental sounds) vary widely in affective content and potentially, other associations. The emotional value of individual bird call stimuli here (as rated by healthy controls) varied quite widely (see Table S1), with the suggestion that certain categories of birds differ in this respect; it remains unclear whether this factor might have modulated BA's responses. More broadly, rather like human vocalisations bird calls serve particular behavioural ends (for example, mating and territorial displays, alarm, imitation) and the semantic processing of these action sounds might therefore engage mechanisms distinct from those engaged in processing other sensory attributes and other categories of sounds (Brefczynski-Lewis and Lewis, 2017).

It should also be acknowledged in this context that the procedures used here to assess BA's ability to attribute meaning to bird calls and to human voices were not identical: whereas bird calls were assessed via a forced choice decision on associated semantic characteristics, human voices were assessed via a forced-choice familiarity decision. These decisions are likely to engage different aspects or levels of semantic processing, leaving open the possibility that BA's differential performance for these auditory categories reflects the response criteria used rather an intrinsic, category-based cognitive dissociation. In principle, it would be possible to equate neuropsychological response procedures for bird calls and voices; however, universal semantic characteristics that could be used to classify famous voices are less straightforward to decide *a priori* than the ethological characteristics we used here to probe bird call knowledge. On the other hand, a familiarity decision on bird calls would be potentially vulnerable to the idiosyncratic exposure of experienced birders to nominally ‘unfamiliar’ birds.

As with any single case study, the present findings do not allow conclusions regarding the neuroanatomical basis for the dissociations observed. It would be of interest to explore the possibility that the mechanism may reside in distinct connectivity profiles mediating the linkage of modality-specific semantic processing to higher-order, multimodal conceptual representations (Martin, 2007, Pulvermüller, 2018) and it would be feasible to explore this using functional neuroimaging techniques in the healthy brain as well as in patients with SD. More broadly, single case studies such as this one have their chief value in illustrating the dissociability and modularity of cognitive functions: to establish the generalisability of our findings to the wider SD population will require complementary studies of patient cohorts.

Taking these caveats into account, the present case provides further evidence that brain knowledge systems subserving environmental sounds have a fractionated organisation and may be differentially targeted by the paradigmatic disorder of semantic memory, SD. The extraordinary richness of avian semantics, paralleled in the auditory and visual (as well as the verbal) modalities, allows a detailed analysis of modality-specific as well as modality-independent effects with respect to this knowledge category. This analysis transcends the effects of acquired expertise and illustrates how single case experiments that address apparently idiosyncratic phenomena can illuminate neuropsychological processes of more general relevance.
