# Supplementary material for: Agnosia for bird calls
Source: Neuropsychologia. 2018 May;113:61–7. doi: 10.1016/j.neuropsychologia.2018.03.024 (PMC5946901; doi:10.1016/j.neuropsychologia.2018.03.024)
Supplement: Supplementary file 1 — Supplementary material [file mmc1.docx]

**Supplementary Material: Agnosia for bird calls, by L Muhammed et al**

**Table S1.** Bird species presented as stimuli in the experimental test of bird knowledge

| **Stimulus category** | **Bird species** | **Pleasantness rating** |
| --- | --- | --- |
| Larger – migratory – water-dwelling | Bittern | 3.5 |
|  | Brent goose | 3 |
|  | Gadwall | 4 |
|  | Goldeneye | 3 |
|  | Oystercatcher | 3.75 |
|  | Shoveler | 4 |
|  | Wigeon | 2.75 |
|  | Woodcock | 3.75 |
|  |  | 3.5 (3 – 4) |
| Larger – migratory – non-water-dwelling | Cuckoo | 4 |
|  | Fieldfare | 3.25 |
|  | Golden oriole | 3.75 |
|  | Honey buzzard | 2.75 |
|  | Hoopoe | 3.25 |
|  | Nightjar | 2.5 |
|  | Redwing | 3.75 |
|  | Turtle dove | 4.25 |
|  |  | 3.4 (2.5 – 4.25) |
| Larger – nonmigratory – water-dwelling | Canada goose | 3 |
|  | Common gull | 2.75 |
|  | Curlew | 3.25 |
|  | Grey heron | 2.75 |
|  | Greylag goose | 2 |
|  | Herring gull | 2.5 |
|  | Lesser black-backed gull | 2.75 |
|  | Mallard | 2.75 |
|  |  | 2.7 (2 – 3.25) |
| Larger – nonmigratory – non-water-dwelling | Barn owl | 1.75 |
|  | Carrion crow | 3 |
|  | Jay | 1.75 |
|  | Kestrel | 3.75 |
|  | Magpie | 2.75 |
|  | Pheasant | 1.75 |
|  | Raven | 2.5 |
|  | Wood pigeon | 3.75 |
|  |  | 2.6 (1.75 – 3.75) |
| Smaller – migratory – water-dwelling | Common sandpiper | 4 |
|  | Golden plover | 2.5 |
|  | Green sandpiper | 3 |
|  | Reed warbler | 3.25 |
|  | Ringed plover | 3 |
|  | Sedge warbler | 3.25 |
|  | Shore lark | 4.75 |
|  | Yellow wagtail | 3.75 |
|  |  | 3.4 (2.5 – 4.75) |
| Smaller – migratory – non-water-dwelling | Blackcap | 4 |
|  | Brambling | 1.75 |
|  | Chiffchaff | 3.5 |
|  | House martin | 3.75 |
|  | Nightingale | 3.75 |
|  | Swallow | 3 |
|  | Swift | 2.75 |
|  | Quail | 3.75 |
|  |  | 3.3 (1.75 – 4) |
| Smaller – nonmigratory – water-dwelling | Cetti’s warbler | 3.5 |
|  | Grey wagtail | 2.5 |
|  | Kingfisher | 3 |
|  | Lapwing | 3.5 |
|  | Pied wagtail | 2.25 |
|  | Redshank | 2.75 |
|  | Reed bunting | 3.25 |
|  | Snipe | 2.75 |
|  |  | 2.9 (2.25 – 3.5) |
| Smaller – nonmigratory – non-water-dwelling | Blackbird | 4 |
|  | Blue tit | 4 |
|  | Chaffinch | 4 |
|  | Dunnock | 4.5 |
|  | House sparrow | 3 |
|  | Robin | 4.25 |
|  | Starling | 3.75 |
|  | Wren | 3.75 |
|  |  | 3.9 (3 – 4.5) |

The stimulus list comprised 64 British bird species likely to be familiar to an experienced amateur birder in the UK. Birds in the list were classified according to each of three key characteristics: size, behaviour (whether or not migratory), habitat (whether or not primarily dwelling near water), based on Royal Society for Protection of Birds data and discussion with an expert ornithological advisor. These categories were then used to arrange stimulus pairs in the final tests of verbal, auditory and visual knowledge of the bird species (see text and Figure 2). Regarding the characteristic of size, ‘large’ and ‘small’ are arbitrary designations while the other categories are not absolute but refer to species’ principal characteristics (the category of ‘water-dwelling’, for example, is subject to exceptions under some circumstances). The key design principle of the test (and the basis for the semantic decision on each trial) was the differential characteristics of the birds comprising each stimulus pair: i.e., we defined species attributes relative to one another (e.g., on trials requiring a size decision, the members of each stimulus pair were chosen such that one bird species was unambiguously larger than the other). Pleasantness ratings on individual bird calls were obtained from four healthy older individuals (two male, two female, aged 58 – 60) who did not participate in the main experiment and do not have birding expertise; ratings were made on a 5-point Likert scale (1, ‘very unpleasant’ to 5, ‘very pleasant’) with the rater blind to the bird species making the sound and the mean score across raters is shown for each bird call.
